# Associations of a liver health index with health, milk yield, and reproductive performance in dairy herds in the northeastern United States

**DOI:** 10.3168/jdsc.2022-0261

**Published:** 2022-09-03

**Authors:** A.L. Kerwin, M.M. McCarthy, W.S. Burhans, D.V. Nydam, S.K. Wall, K.M. Schoenberg, K.L. Perfield, T.R. Overton

**Affiliations:** 1 Department of Animal Science, Cornell University, Ithaca, NY 14853; 2 Adisseo USA Inc., Alpharetta, GA 30022; 3 Dairy-Tech Group, South Albany, VT 05875; 4 Department of Population Medicine and Diagnostic Sciences, College of Veterinary Medicine, Cornell University, Ithaca, NY 14853; 5 Elanco US Inc., Greenfield, IN 46140

## Abstract

The objective was to evaluate a liver health index (LHI) by evaluating its association with negative health events, milk yield, and risk of pregnancy within 150 d in milk (DIM). In a retrospective cohort study, an LHI was calculated based on plasma albumin, cholesterol, and bilirubin concentrations for 265 primiparous and 611 multiparous cows 3 to 12 DIM enrolled across 72 farms in the northeastern United States. Mixed effects linear regression models were used to evaluate if (1) metritis (MET), (2) displaced abomasum (DA), (3) clinical ketosis (CK), (4) one or more of the 3 disorders (MET, DA, or CK), (5) 2 or more of the 3 disorders (MET, DA, or CK), or (6) culling within 30 DIM was associated with LHI. Mixed effects linear regression models were used to evaluate if LHI was associated with 305-d mature equivalent milk at the fourth test day (ME305; mean ± standard deviation: 114 ± 13 DIM) and a Cox proportional hazards model was used to evaluate if LHI was associated with pregnancy within 150 DIM. Cows that were diagnosed with MET, DA, CK, one or more of the disorders, 2 or more of the disorders, or were culled within 30 DIM had a lower LHI than cows that were not diagnosed with a disorder or culled. A 1-unit increase in LHI was associated with a 154 ± 38 kg increase in ME305 and a 8% increased risk of pregnancy within 150 DIM [hazard ratio (95% confidence interval): 1.08 (1.03 to 1.14)] for multiparous cows; however, we did not identify a relationship between LHI and ME305 or pregnancy within 150 DIM for primiparous cows. These results suggest that the LHI is associated with health, milk yield, and pregnancy within 150 DIM for multiparous cows and health for primiparous cows; therefore, the LHI can be used as a tool to evaluate transition cow success.

## Highlights

• The early postpartum cow is characterized as being in an increased inflammatory state.
• Integrating multiple biomarkers into an index may be useful for assessing transition cow success.
• The LHI was associated with health, milk, and reproduction.
• Our LHI may be useful for evaluating transition cow success.

## Graphical Abstract

**Figure:**
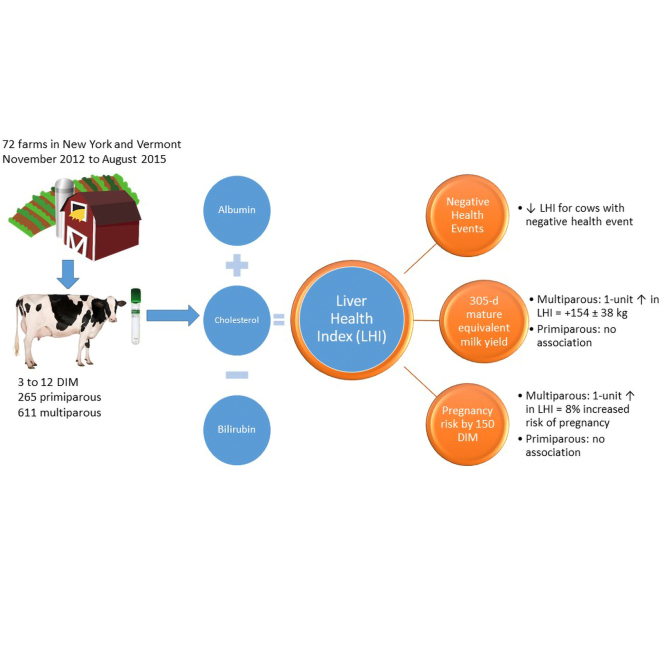
**Summary:** Existing liver biomarker indices used to evaluate transition cow health and inflammatory status utilize multiple blood samples across the first month of lactation, which may not provide timely information for identifying subclinical cases and may be financially prohibitive. Our objective was to evaluate a liver health index (LHI), which integrates albumin, cholesterol, and bilirubin values from 1 early lactation plasma sample, by evaluating the association with negative health events, milk yield, and pregnancy within 150 days in milk (DIM). The LHI was calculated for 876 cows, 3 to 12 DIM, across 72 farms. Cows diagnosed with any negative health event had a lower LHI than cows not diagnosed with a negative health event. We identified a positive relationship between LHI and milk yield and pregnancy within 150 DIM for multiparous cows; however, LHI was not associated with milk yield or pregnancy within 150 DIM for primiparous cows.

The early postpartum cow is characterized as being in an increased inflammatory state due to the act of parturition, dietary changes resulting in gastrointestinal hyperpermeability, infectious or metabolic diseases, and environmental stressors (Trevisi and Minuti, 2018; Horst et al., 2021). Identifying blood biomarkers associated with inflammation can aid in identifying cows that may need attention, particularly subclinical cases; however, identifying a single biomarker may not be ideal due to the complexity of the immune system response and challenges with interpreting the results (Bertoni and Trevisi, 2013). Integrating multiple biomarkers into an index may improve the ease of interpretation as these biomarkers may not change uniformly when evaluating different negative health disorders (Ceciliani et al., 2012). Albumin (**Alb**), cholesterol (**Chol**), and total bilirubin (**Bili**) are negative acute phase proteins or related parameters that can indicate liver function and inflammatory status. Blood Alb concentrations decrease as the positive acute phase protein response is initiated by proinflammatory cytokines (Bradford et al., 2015). Blood Chol concentrations are used as a proxy for lipoproteins originating from the intestine and liver (Bertoni and Trevisi, 2013). Total Bili concentrations are measured as a proxy for the liver enzymes responsible for clearing Bili (Farid et al., 2013; Trevisi and Minuti, 2018).

Bertoni and Trevisi (2013) reviewed 2 indices utilizing different biomarkers for evaluating transition cow health: the liver activity index (**LAI**; Trevisi et al., 2001; Bertoni et al., 2008) and the liver functionality index (**LFI**; Bertoni et al., 2006; Trevisi et al., 2012). The LAI evaluates blood concentrations of negative acute phase reactants (Alb, Chol, and retinol) of an individual at 7, 14, and 28 DIM and is calculated relative to the sampling population. The LAI is most useful for ranking cows within a herd; however, since the individual cow values are based on the mean and standard deviation of the sampled population, there is a greater chance of misclassification. The LFI evaluates blood concentrations of negative acute phase reactants (Alb, Chol, and Bili) of an individual at 3 and 28 DIM. Since this calculation is standardized to a known healthy cow, the LFI is useful for comparing cows across herds or within a herd. Both indices were associated with an inflammatory response such that a lower LAI or LFI indicated a more severe inflammatory response (Bertoni and Trevisi, 2013). These indices may be used to determine if a cow successfully transitioned to lactation; however, it may not be practical for dairy producers to wait until at least 28 DIM to identify cows that may not have successfully transitioned. In addition, it can be costly to measure retinol and sample cows multiple times. Developing an index that requires sampling a cohort of cows once early in lactation within a narrow range of DIM would provide dairy producers a tool for early detection of cows that are adapting poorly to lactation and for herd level monitoring purposes.

The objective was to evaluate a liver health index (**LHI**) by evaluating the association with negative health events, milk yield, and pregnancy within 150 DIM. We hypothesized that cows with a negative health event would have a lower LHI compared with cows without the negative health event and an increase in LHI would be associated with increased milk yield and risk of pregnancy within 150 DIM.

A more in-depth description of the study population and study design was previously published (Kerwin et al., 2022b). Briefly, a retrospective cohort study was conducted from a convenience sample of 72 farms located in New York and Vermont between November 2012 and August 2015. All procedures involving cows in this study were approved by the Cornell University Institutional Animal Care and Use Committee, protocol #2012–0124. Inclusion criteria for herds were (1) Holstein herds, (2) ≥400 milking cows, (3) freestall housing, (4) TMR-fed herds, and (5) enrolled in monthly DHI testing or have on-farm milk recording with record management by Dairy Comp 305 (DairyComp 305, Valley Ag Software) or PCDART (PCDART, Dairy Records Management System).

One 10-mL blood sample was collected from 877 cows at a single time point between 3 to 12 DIM (4 to 20 cows per farm) into a sodium-heparin tube from the coccygeal vessels (Vacutainer, Becton Dickinson). Samples were stored on ice until processing within 6 h of collection. Samples were centrifuged at 1,350 × *g* for 15 min at room temperature using a tabletop centrifuge, and plasma was separated and stored at −20°C. Plasma samples were analyzed for Alb, Chol, and Bili on a Roche Cobas 6000 c501 biochemistry analyzer using commercially available kits (Roche Diagnostics) at the University of Guelph Animal Health Laboratory (Ontario, Canada).

Modified from the LAI and the LFI by Bertoni and Trevisi (2013), an LHI was calculated for each cow using the individual's Alb, Chol, and Bili values and the sampled population mean and standard deviation using the following equation (previously described as the metabolite health index; Gallagher et al., 2019):

LHI = [(Alb − μ_Alb_)/σ_Alb_] + [(Chol − μ_Chol_)/σ_Chol_] − [(Bili − μ_Bili_)/σ_Bili_],

where µ = the overall sampling population mean and σ = the overall sampling population standard deviation. Health event records, reproductive records, and milk records were acquired through the farm's record management software program when all sampled cows were at least 150 DIM. A more complete description of health disorder definitions were described previously (Kerwin et al., 2022b).

A sample size calculation was conducted to estimate the prevalence of cows with hyperketonemia within a farm for a prospective cohort study previously described by Kerwin et al. (2022a). Raw data were entered into Microsoft Excel (Microsoft Corp.) and data cleaning was conducted before data analysis to correct human data recording errors.

All statistical analyses were calculated using SAS software (SAS 9.4, SAS Institute Inc.). With cow as the experimental unit, linear mixed regression models using PROC MIXED and farm as a random effect were developed to evaluate if farm recorded (1) metritis (**MET**), (2) displaced abomasum (**DA**), (3) clinical ketosis (**CK**), (4) one or more disorder (MET, DA, and CK), (5) 2 or more disorders (MET, DA, and CK), or (6) culling within 30 DIM was associated with LHI. A farm was removed from the analysis if the disorder was not recorded. Cows with missing covariate information were not included in the analysis unless if the covariate was not included in the final model. Covariates were first tested using a simple linear mixed regression model to evaluate the association with LHI. Covariates were included in the full model if *P* < 0.20. Covariates considered (described further in Kerwin et al., 2022b) were parity group (primiparous vs. multiparous), calving difficulty (**CD**; calving score <3 vs. calving score ≥3 or twins), locomotion score at the time of blood sample collection (**LS**; <3 vs. ≥3), calving season [cool (October through April) vs. warm (May through September)], BCS at close-up visit (−18 to −4 d relative to parturition; ≤3.25 vs. >3.25) and fresh visit (3 to 12 DIM; ≤3.0 vs. >3.0), and the change in BCS from the close-up to fresh visit (≥−0.25 vs. <−0.25). Days in milk at the time of blood sampling was forced into the model as a covariate. Multicollinearity between covariates included in the full model was assessed using PROC REG and determined to be present if a covariate had a tolerance <0.40 (Allison, 2012). If multicollinearity was identified between 2 covariates, the covariate with the greatest *P*-value from the univariate analysis was removed and multicollinearity was reassessed. Manual backward stepwise elimination was used to remove variables with *P* ≥ 0.05.

With cow as the experimental unit, mixed linear regression models using the MIXED procedure and farm as a random effect were developed to evaluate 305-d mature equivalent milk yield at approximately the fourth test day (**ME305**). Primiparous and multiparous cows were analyzed separately. Farms and cows were removed from the analysis if ME305 was not recorded. Cows with missing covariate information were not included in the analysis unless if the covariate was not included in the final model. A simple linear regression (PROC MIXED) was used to test potential covariates to include in the initial ME305 full model, which included LS, BCS at the fresh period visit, change in BCS from the close-up visit to the fresh visit, and calving season. Variables were included in the full model if *P* < 0.20 and a manual backward stepwise elimination was used to remove variables with *P* ≥ 0.05 while forcing the main effect of LHI in the model. Multicollinearity was assessed, as described previously.

With cow as the experimental unit, a Cox proportional hazards model using the PHREG procedure and farm as a random effect were developed to evaluate pregnancy within 150 DIM. Cows that died or were sold before the end of the herd's voluntary waiting period were not included in the analysis as they were never eligible to be bred. Cows that left the herd after the voluntary waiting period or not pregnant by 150 DIM were right-censored. Cows with missing covariate information were not included in the analysis unless if the covariate was not included in the final model. Primiparous and multiparous cows were analyzed separately. Potential covariates were assessed using a univariate analysis and if the association between the covariate and pregnancy was *P* < 0.2, then it was offered to the model and manual backward stepwise elimination was used to remove any variable with *P* ≥ 0.05 while forcing the main effect of LHI in the model. Covariates tested included ME305 (above vs. below median production of the sampled cohort within each herd), LS, BCS at the fresh period visit, change in BCS from the close-up visit to the fresh visit, CD, and calving season. The proportional hazards assumption was checked statistically for the time-dependent covariates (Allison, 2010) and a sensitivity analysis was performed to evaluate noninformative censoring. If the proportional hazards assumption was violated, a time-dependent variable was included in the model and a Wald test was performed in 10 DIM increments from 50 to 150 DIM (Allison, 2010).

The distribution of plasma analytes is reported in Table 1. One cow was removed from the analysis due to the plasma sample having moderate hemolysis, as defined by the laboratory where analysis was performed, leaving 265 primiparous and 611 multiparous cow for the analysis. For the MET analysis, 5 farms were removed due to not recording the disorder (n = 58 cows) and 25 cows were not included in the final model due to missing covariate information. For the CK analysis, 1 farm was removed due to not recording the disorder (n = 8 cows) and 35 cows were not included in the final model due to missing covariate information. For the one or more disorder model and 2 or more disorders model, 6 farms were removed due to not recording one of the disorders (n = 66 cows) and 25 cows were not included in the final model due to missing covariate information. All farms recorded culling within 30 DIM and DA; however, 35 cows were not included in the final model due to missing covariate information. For the ME305 models, 2 farms (n = 2 primiparous, 15 multiparous), 19 primiparous cows, and 64 multiparous cows were removed due to missing ME305 data, and 3 multiparous cows were not included in the final model due to missing covariate information. For the pregnancy within 150 DIM models, farms were removed due to using natural service or because the farm's reproductive management was altered during the period of data collection (n = 3 farms representing 6 primiparous and 21 multiparous cows) and individual cows from remaining farms were removed if they were culled before the end of the farm's VWP (n = 12 primiparous and 29 multiparous).

**Table 1:** Distribution of plasma analytes and the calculated liver health index (LHI) for samples obtained from 876 Holstein cows (n = 265 primiparous, 611 multiparous) that were 3 to 12 DIM from 72 farms across the northeastern United States

Analyte	Mean	Median	SD	IQR^1^	Range
Albumin, g/L	30.79	31.0	3.87	28.0 to 33.0	18.0 to 55.0
Cholesterol, mmol/L	2.25	2.20	0.63	1.80 to 2.66	0.55 to 6.31
Bilirubin, μmol/L	1.66	1	1.50	1 to 2	0 to 11
LHI^2^	0	0.11	2.14	−1.29 to 1.42	−8.80 to 8.61
LHI primiparous	−0.09	0.06	1.81	−1.22 to 1.14	−8.80 to 3.84
LHI multiparous	0.04	0.13	2.27	−1.31 to 1.55	−8.06 to 8.61

1 IQR = interquartile range.

2 LHI (arbitrary units) = [(Alb − μ_Alb_)/σ_Alb_] + [(Chol − μ_Chol_)/σ_Chol_] − [(Bili − μ_Bili_)/σ_Bili_], where Alb is albumin, Chol is cholesterol, Bili is bilirubin, μ = the overall sampling population mean, and σ = the overall sampling population standard deviation.

Cows diagnosed with any negative health event was associated with a lower LHI compared with cows not diagnosed with the negative health event of interest (Table 2; *P* < 0.001). Other covariates that remained in all models included BCS at the fresh visit, BCS change, LS (except culling model), and CD. Overall, cows that had a fresh period BCS >3.0, lost less BCS (BCS change ≥−0.25), were not lame (LS <3), or did not have a difficult calving (calving score <3) had a higher LHI than their counterparts. As DIM at time of blood sample collection increased, LHI was associated with an increase of 0.21 ± 0.03 units/d (*P* < 0.001). Multicollinearity was not identified within any model.

**Table 2:** Least squares means and SEM of the liver health index for 6 linear mixed models for cows diagnosed or not diagnosed with the negative health event of interest for a retrospective cohort study involving 72 farms across the northeastern United States

Variable^1^	n (%)		LSM		SEM	*P*-value
	Disorder	No disorder	Disorder	No disorder		
MET	71 (9.0)	722 (91.1)	−1.79	−0.54	0.27	<0.001
DA	23 (2.7)	818 (97.3)	−3.23	−0.57	0.42	<0.001
CK	72 (8.6)	761 (91.4)	−2.22	−0.50	0.26	<0.001
Cull	27 (3.2)	814 (96.8)	−3.55	−0.55	0.38	<0.001
Any of 3	132 (16.8)	653 (83.2)	−1.97	−0.35	0.21	<0.001
2 or more	25 (3.2)	760 (96.8)	−3.05	−0.58	0.41	<0.001

1 MET = metritis; DA = displaced abomasum; CK = clinical ketosis; Cull = culling within 30 DIM; Any of 3 = 1 or more of DA, CK, and MET; 2 or more = 2 or more of DA, CK, and MET.

We did not identify an association between the LHI and ME305 for primiparous cows (*P* = 0.90; n = 239). For the multiparous cow model, every 1-unit increase in LHI was associated with a 154 ± 38 kg increase in ME305 milk (*P* < 0.001; n = 529) and cows that lost less BCS (≥−0.25) was associated with 419 ± 197 kg more ME305 milk than cows that lost more BCS (<−0.25). Multicollinearity was not identified within any model.

For the pregnancy within 150 DIM models, 29.6% (n = 73/247) and 38.9% (n = 218/561) of primiparous and multiparous cows were right-censored, respectively. While controlling for ME305 (*P* = 0.006), we did not identify an association between the LHI and pregnancy within 150 DIM for primiparous cows (*P* = 0.68). The proportional hazards assumption was violated for the multiparous cow model; however, when including the interaction between time and LHI, the interaction term had a *P* = 0.19 and was therefore removed and the average effect of index over the 150 DIM is reported. Multiparous cows were associated with an 8% increased risk of pregnancy within 150 DIM for every 1-unit increase in LHI [hazard ratio (95% CI): 1.08 (1.03 to 1.14); *P* = 0.002]. No covariates remained in the multiparous cow model (*P* > 0.05).

Our study assessed an LHI by evaluating the association with negative health events, ME305 milk, and pregnancy within 150 DIM. Cows that were diagnosed with MET, DA, CK, one or more disorder, 2 or more disorders, or were culled within 30 DIM were associated with a lower LHI than cows that were not diagnosed with a negative health disorder or culled. The LHI was positively associated with milk yield and pregnancy within 150 DIM for multiparous cows; however, we did not observe a relationship for primiparous cows for either outcome, which may be attributed to different homeorhetic mechanisms (Bauman and Currie, 1980). Primiparous cows tend to have a slower rise in milk yield, resulting in an initial lower demand of nutrients for milk yield compared with multiparous cows. Therefore, compared with multiparous cows, more glucose and nutrients may be available and spared for the inflammatory response, allowing primiparous cows to resolve the inflammation quickly without inhibiting future milk and reproductive performance. Differences between primiparous and multiparous cows may also be attributed to the functional decline of the immune system as age increases, also known as immunosenescence (Weiskopf et al., 2009; Buggiotti et al., 2021).

In a study with 120 multiparous cows, Bertoni et al. (2008) reported cows in the lower quartile for the LAI versus the upper quartile had an increased frequency of negative health disorders and lower milk yield. Results evaluating reproductive performance are mixed; however, overall, cows in the upper quartile had the lowest number of services per pregnancy (*P* = 0.12), days open (*P* = 0.02), percent of repeat breeders (>3 inseminations per pregnancy; *P* = 0.10), and the highest probability of pregnancy (*P* = 0.22) compared with cows in the upper intermediate, lower intermediate, and low quartiles. Bossaert et al. (2012) retrospectively dichotomized high-yielding cows (n = 21) into low (−0.51 ± 0.08) or high (0.45 ± 0.12) LAI groups and reported an association between disease incidence and LAI group (*P* = 0.024) such that the low LAI group had greater incidence (n = 6/10) than the high LAI group (n = 1/11).

In a 54 multiparous cow study, Trevisi et al. (2012) compared the 6 cows with the highest LFI to the 6 cows with the lowest LFI. The authors reported more cases of health problems (13 vs. 3) and lower milk yield within the first month of lactation (35.5 vs. 40.4 kg/d; *P* = 0.07) in the low LFI cows versus the high LFI cows. Zhou et al. (2016) reported no clinical cases of disorders in an 18 multiparous cow study evaluating LFI; however, the authors reported lower milk yield from d 14 to 28 of lactation in low LFI cows (LFI <0; n = 10) versus high LFI cows (LFI >0; n = 8). Though caution should be used when interpreting health events due to a limited sample size, Zhou et al. (2017) reported a greater incidence of negative health disorders, such as ketosis and DA, in low LFI cows (LFI <0; n = 9 multiparous cows) versus high LFI cows (LFI >0; n = 31 multiparous cows). The authors also reported lower milk yield in the first month of lactation for low LFI cows versus high LFI cows (36.2 vs. 46.3 kg/d). Another study evaluated the LFI in clinically healthy cows on a commercial dairy farm in Brazil (Montagner et al., 2016). The authors classified 20 multiparous (≥3 lactation) as low LFI (LFI = −12 to −7; n = 10) or high LFI (LFI = −7 to −4; n = 10) and reported resumption of normal ovarian activity in the first 7 wk of lactation in a lower proportion of low LFI cows (n = 3/10) compared with high LFI cows (n = 9/10; *P* < 0.05); however, there was no evidence that milk yield differed between the 2 groups through 60 DIM.

The previous studies discussed only evaluated multiparous cows on ≤3 farms. To our knowledge, this is the first study identifying associations between a liver biomarker index in primiparous cows with health, milk yield, and reproductive outcomes. In addition, our study evaluates the LHI on a large number of dairy farms with a wide variety of management and nutritional styles (Kerwin et al., 2022b). Our study design aimed to evaluate long-term effects on milk yield and reproductive performance, whereas most of the previous studies discussed evaluated short-term milk yield effects (<60 DIM). Future work should investigate the short-term effects of LHI on milk yield and reproductive performance, particularly for primiparous cows.

To conclude, the results of this study suggest improved postpartum performance (decreased disorder incidence, increased milk yield, and increased risk of pregnancy within 150 DIM) for cows with a greater LHI; however, LHI was not associated with milk yield or risk of pregnancy within 150 DIM for primiparous cows. Our results corroborate the results of previous studies evaluating the LAI and LFI; therefore, the results of our study validate the use of the LHI for evaluating transition cow success. Evaluating the LHI in a single blood sample early in lactation as a means for evaluating a herd's transition cow program is financially beneficial, can identify potentially problematic cows earlier in lactation, and can aid in decision-making versus indices that require multiple time point samples. We envision the LHI equation can be applied using 2 general approaches: (1) samples can be taken within a herd or across herds and the LHI calculated using the sampling population means and standard deviations to rank cows in the population or (2) samples from an individual or herd can be compared with our study population by calculating the LHI with our reported means and standard deviations to evaluate transition cow success; though for direct comparison, plasma samples should be analyzed as reported in this study due to assay variation.
